# Use of Complementary and Alternative Medicine in Japan: A Cross-sectional Internet Survey Using the Japanese Version of the International Complementary and Alternative Medicine Questionnaire

**DOI:** 10.31662/jmaj.2018-0044

**Published:** 2019-03-04

**Authors:** Yoshiharu Motoo, Keiko Yukawa, Ichiro Arai, Kazuho Hisamura, Kiichiro Tsutani

**Affiliations:** 1 Department of Medical Oncology, Kanazawa Medical University, Uchinada, Japan; 2 Department of Health Policy and Technology Assessment, National Institute of Public Health, Wako, Japan; 3 Department of Pharmaceutical Sciences, Nihon Pharmaceutical University, Kita-adachigun, Japan; 4 Faculty of Health Sciences, Tokyo Ariake University of Medical and Health Sciences, Tokyo, Japan

**Keywords:** Complementary and alternative medicine (CAM), I-CAM-Q, Japan, Kampo, internet survey

## Abstract

**Introduction:**

Although there have been several national survey studies on complementary and alternative medicine (CAM) use in Japan, previous studies have not been compared with investigations conducted in other countries. An international CAM questionnaire known as I-CAM-Q was developed through a two-day international workshop in 2006. The purpose of this study was to investigate the use of CAM by the general Japanese population using a modified version of the I-CAM-Q for the Japanese (I-CAM-QJ).

**Methods:**

We developed the I-CAM-QJ to conduct an internet survey of 3,208 participants from the general population of Japan in February 2016. The respondents included 1,592 males (49.6%), 1,348 university graduates (38.8%), 1,105 individuals in good health (34.4%), and 1,028 individuals with long-term illness or disability (32.0%).

**Results:**

Of the 3,208 respondents, 411 participants reported CAM use during the past 12 months (12.8%). The following therapies and products were used: Kampo medicines (over-the-counter Kampo medicines: 15.7%; prescribed Kampo medicines: 15.4%), dietary supplements 11.8%, massage services 3.9%, and physical therapy 3.5%. Regarding the use of self-care methods during the last 12 months, the following methods and products were used: bath salts 25.8% and walking 25.3%.

**Conclusions:**

An internet survey on CAM use by the general Japanese population with a modified I-CAM-Q (I-CAM-QJ) revealed that Kampo medicines and dietary supplements were the most commonly used CAMs in Japan.

## Introduction

The use of complementary and alternative medicine (CAM) is increasing in developed countries along with changes in the morbidity and mortality caused by diseases and increased individual health awareness. For example, in the United States, CAM has been an object of academic research since the 1990s ^(1)^, and the National Center for Complementary and Integrative Health (NCCIH) is currently examining this phenomenon ^(2)^.

In Japan, Kampo medicines (traditional Japanese herbal medicines), acupuncture, moxibustion, massage and dietetics have been used since the early Edo period (17th century) as “authentic medicine”. Currently, 148 Kampo formulas are covered by the national health insurance. In addition to Kampo medicines, it is reported that the use of dietary supplements as CAM is also increasing in Japan ^(3), (4), (5), (6), (7)^. However, previous studies on Kampo medicines uses in Japan were not comparable with those conducted in other countries although some characteristics of CAM use have been revealed through these reports. This suggests that a new measure is needed with international comparability.

The International Complementary and Alternative Medicine Questionnaire (I-CAM-Q) was developed since a two-day workshop in Sommarøy, Norway, in September 2006 ^(8)^. The participants of the meeting were not solely from Europe, but from the USA and other countries, and the questionnaire was not planned as a European tool but as an international tool. Several studies on CAM use have been reported using translated and modified versions of I-CAM-Q ^(9), (10), (11)^.

This study aimed to clarify the prevalence of CAM use by the general Japanese population using the Japanese version of the I-CAM-Q (I-CAM-QJ). In this study, we defined CAM as a group of diverse medical and health care systems, practices, and products that are not considered to be a part of conventional healthcare, following a report by Kristoffersen AE et al. ^(12)^. We included Kampo Medicine in CAM because it originates from ancient Chinese medicine ^(13)^. Chinese Medicine is internationally considered to be one of CAM.

## Materials and Methods

### 1. Development of the Japanese version of the I-CAM-Q (I-CAM-QJ)

The English version of the I-CAM-Q was translated into Japanese and modified to meet conditions in Japan. The original structure of the I-CAM-Q, composing of the following four parts, was maintained. Part 1: From whom were the medical/health services received? Part 2: What sort of CAM-related recommendations have you received from your physician? Part 3: Use of orally administered products. Part 4: Use of self-care methods. In addition, the I-CAM-Q ascertains the following for each parts: 1) presence of use, 2) the main reason for use, and 3) how helpful the therapy/product was.

We discussed and made some changes in each of the four parts of the I-CAM-Q. In Part 1, we deleted herbalists, who are very uncommon in Japan, and added acupuncture/moxibustion specialists, who are board-certified, and yoga instructors, who are not in the national certification system. Regarding Part 2, “CAM provided by physicians” was reworded as “CAM with a doctor’s prescription or advice.” The rewording was done because of the unique characteristics of certain CAM modalities in Japan. As for acupuncture and moxibustion, it is uncommon for Japanese physicians to practice acupuncture themselves, while physicians write diagnostic certificates for national health insurance-covered acupuncture and moxibustion for patients with several diseases and conditions, such as low back pain. Physicians also do not sell dietary supplements, instead only advice patients about their use. In Part 3, we classified orally administered CAM into four categories: 1) dietary supplements, 2) Kampo products, 3) homeopathy, and 4) diet therapy. In Part 4, we added as many self-care or self-help items as possible, reflecting recent situations in Japan.

A pilot interview survey was conducted using a draft modified questionnaire on the use of CAM at three sites, i.e., Kanazawa (oncology outpatient clinic of a university hospital), Kyoto (hot spring facilities) and Tokyo (private clinic), with 20 participants for each site. Variations in the definition, purpose of use, the presence of a CAM practitioner, and survey period (length of the recall period) were observed. The final version of the I-CAM-QJ in English is shown in Supplementary Material 1.

### 2. Survey participants

The targeted sample size was 3,200 participants residing in Japan. Internet survey requests were sent to 11,566 participants by random extraction from approximately 1.4 million participants with the help of the survey company Intage, Inc. (Tokyo, Japan). For each age group, i.e., 20-29, 30-39, 40-49, 50-59, and 60-69 years, approximately 300 samples were needed from both males and females to achieve the target sample size (300 X 2 X 5=3,000). The survey implementation date was March 16, 2016. Healthcare professionals and media people were excluded. There was no stratification for the region of the country.

### 3. Surveys

Requests to complete the internet survey were sent via e-mail. The questionnaire was e-mailed again to groups that did not achieve the target number of 300 by the deadline. The total number of participants was 11,568 after two requests. Survey requests were stopped at 9:00 on March 18, 2016, because the number of respondents (3,208) exceeded 3,000, which was the target number for the survey.

### 4. Explanation of survey items

The explanation for several of the surveyed therapies are provided below.

Physical therapy: manipulation methods using the hands or elbows, such as relaxation techniques for muscles and pelvic correction. Chiropractic: manipulation correcting the curve of the spine. Massage/shiatsu: a form of manipulation by thumbs, fingers, and palms without the use of instruments to apply pressure to the human skin to correct internal malfunctions. Heat therapy: treatment by the application of heat caloric stimulation (high-frequency hyperthermia). Magnetic therapy: treatment using high-strength magnetic force lines generated by permanent magnets housed within a personal accessory magnetic device. Homeopathy: a system of treating diseases in which sick people are given very small amounts of natural substances that would produce the same effects as the diseases produce in healthy people.

Kampo medicines prescribed by physicians as well as over-the-counter (OTC) Kampo medicines were regarded as CAM. Kampo medicine originates from ancient Chinese medicine but has been adapted to Japanese culture for more than 1,000 years ^(13)^. Prescribed Kampo medicines are approved as legal pharmaceutical products and are covered by the national health insurance in Japan. The characteristics of each Kampo formula can be found on the Standards of Reporting Kampo Products website (STORK, http://mpdb.nibiohn.go.jp/stork/) managed by a Japanese national institute known as the National Institutes of Biomedical Innovation, Health and Nutrition (NIBIOHN) ^(14)^.

Regarding self-care, in addition to the basic items of the I-CAM-Q questionnaire, we included a total of 20 items with a high proportion of use in previous studies in Japan ^(15)^. As for walking in self-care methods, walking in this survey was based on a person’s will and not prescribed as an exercise therapy. Walking is a commonly used CAM, especially in East Asian countries, such as China ^(16)^ and Korea ^(17)^.

### 5. Ethical considerations

The study received ethics approval by the Ethics Committee of the Tokyo Ariake University of Medical and Health Sciences was obtained (approval no.168 on 21 July 2015). All personal information was omitted from the survey, and data obtained from the survey company did not contain any personal information.

## Results

### 1. Participants characteristics

Responses were obtained from a total of 3,208 participants, with approximately 300 respondents for both males and females, ranging in age from 20 to 69 years with the mean age of 46.4 years (Table 1-1). We stopped the recruitment at the time when the response number exceeded the target number of approximately 3,000. The participants included 1,592 (49.6%) males, 1,348 (38.8%) university graduates, 1,105 (34.4%) individuals in good health, 1,379 (43.0%) individuals in tolerable health, and 1,028 (32.0%) individuals with long-term illness or disability. Specifically, 240 (23.3%) had hypertension, 178 (17.3%) suffered from mental disorders, and 139 (13.5%) had musculoskeletal diseases (Table 1-1).

**Table 1-1.** Participants Demographics/Characteristics (n=3,208).

	N	%	%
Gender			
Male	1,592	49.6	
Female	1,616	50.4	
Age cohort			
20s	633	19.7	
30s	638	19.9	
40s	644	20.1	
50s	650	20.3	
60s	643	20.0	
Final Education			
Junior High School	86	2.7	
High School	1,019	31.8	
Technical School	340	10.6	
Junior College	35	10.8	
University	1,348	38.8	
Graduate School	124	4.1	
Health status			
Very good	135	4.2	
Good	1,105	34.4	
Tolerable	1,379	43.0	
Not very good	526	16.4	
Very poor	63	2.0	
Long-term disease/disability			
Yes	1,028	32.0	
		% in 1,028	% in 3,208
Hypertension	240	23.3	7.5
Mental diseases	178	17.3	5.5
Musculoskeletal diseases	139	13.5	4.3
Skin diseases	133	12.9	4.1
Hyperlipidemia	125	12.2	3.9
Eye diseases	111	10.8	3.5
Diabetes mellitus	99	9.6	3.1
Digestive diseases	88	8.6	2.7
Nasal diseases	88	8.6	2.7
Dental diseases	83	8.1	2.6
Respiratory diseases	67	6.5	2.1
Cardiovascular diseases	43	4.2	1.3
Cancer/malignant diseases	36	3.5	1.1
Renal/urological diseases	35	3.4	1.1
Ear diseases	34	3.3	1.1
Immunological diseases	26	2.5	0.8
Trauma	15	1.5	0.5
Stroke	14	1.4	0.4
Hematological diseases	14	1.4	0.4
Others	159	15.5	5.0

Of the 3,208 respondents, 411 participants reported CAM use during the past 12 months (12.8%). The prevalence of CAM use in each attribute is shown in Table 1-2. CAM was most frequently used in females in their 40s and 50s, with a university level of education and the tolerable chronic diseases described above, and in therapies covered by the national health insurance in 307 out of 411 (69.6%).

**Table 1-2.** Subject Attributes of User of CAM Prescribed and/or Advised by Physicians (n=411).

	N	% in 411	% in 3,208
Gender			
Male	181	41.0	5.6
Female	230	52.2	7.2
Age cohort			
20s	77	17.5	2.4
30s	64	14.5	2.0
40s	82	18.6	2.6
50s	91	20.6	2.8
60s	97	17.5	3.0
Final Education			
Junior High School	15	4.2	0.5
High School	111	31.4	3.5
Technical School	41	11.6	1.3
Higher professional school	3	0.8	0.1
University	165	46.6	5.1
Graduate School	19	5.4	0.6
Health status			
Very good	8	1.9	0.2
Good	129	31.4	4.0
Tolerable	163	39.7	5.1
Not very good	100	24.3	3.1
Very poor	11	2.7	0.3
Long-term disease/disability			
Yes	207	46.9	
		% in 207	% in 3,208
Hypertension	51	24.6	1.6
Mental diseases	44	21.3	1.4
Musculoskeletal diseases	40	19.3	1.2
Skin diseases	29	14.0	0.9
Hyperlipidemia	26	12.6	0.8
Eye diseases	20	9.7	0.6
Diabetes mellitus	24	11.6	0.7
Digestive diseases	19	9.2	0.6
Nasal diseases	18	8.7	0.6
Dental diseases	20	9.7	0.6
Respiratory diseases	16	7.7	0.5
Cardiovascular diseases	10	4.8	0.3
Cancer/malignant diseases	7	3.4	0.2
Renal/urological diseases	8	3.9	0.2
Ear diseases	10	4.8	0.3
Immunological diseases	10	4.8	0.3
Trauma	5	2.4	0.2
Stroke	2	1.0	0.1
Hematological diseases	3	1.4	0.1
Others	36	17.4	1.1

### 2. Providers of medical and healthcare services

Table 2 presents the responses to the question “From whom did you receive medical and healthcare services?” Physicians accounted for the majority of responses at 54.8% (1,757 out of 3,208). However, this question was for medical and health care services (including not only CAM but also conventional medicine). Dentists (34.9%), pharmacists (29.4%), and nurses (18.5%) are healthcare professionals that also provide conventional medicine/healthcare and/or CAM. CAM practitioners included massage/shiatsu practitioners (5.7%), physical therapists (4.9%), judo therapists (3.1%), and acupuncture/moxibustion practitioners (2.6%).

**Table 2.** Healthcare Providers Visited by the Participants during the Last 12 Months (n=3,208).

	Practitioner/Provider	n	%
1	Physician	1,757	54.8
2	Dentist	1,119	34.9
3	Pharmacist	942	29.4
4	Nurse	595	18.5
5	Massage/Shiatsu practitioner	184	5.7
6	Physical therapist	156	4.9
7	Judo therapist	100	3.1
8	Acupuncture/Moxibustion practitioner	84	2.6
9	Midwife	69	2.2
10	Yoga instructor	63	2.0
11	Nutritionist	34	1.1
12	Chiropractor	33	1.0
13	Aromatherapist	29	0.9
14	Spiritual therapist	7	0.2
15	Homeopathy therapist	2	0.1
16	Others	34	1.0
18	No health services or CAM received during the last 12 months	1,030	32.1

### 3. Use of CAM

Individuals who received medical and healthcare from physicians (n = 1,757) were asked what kind of CAM they used. A “CAM prescribed by a physician” is the CAM used by the general population with the advice or recommendation of a physician. The use of prescribed Kampo medicines was reported by 9.2% of respondents, and OTC Kampo medicine use was reported by 3.9% of respondents (Table 3-1). “CAM not provided by a physician” is the CAM used by the general population without any advice or recommendation from a physician. This questionnaire was administered to all respondents (n=3,208). Dietary supplements (11.8%), massage (3.9%), and OTC Kampo medicines (3.5%) were major CAMs in this category (Table 3-2). We classified CAM into three types, namely, the "product-type" (25.0%), "practitioner-type" (7.9%), and "self-care type" (6.5%), although there were CAMs with multiple or overlapping uses.

**Table 3-1.** CAM Provided/Prescribed by Physicians (n = 1,757) ^a)^.

	Product/Treatment Method	N	%
1	Prescribed Kampo medicines	162	9.2
2	Over-the-counter Kampo medicines	69	3.9
3	Dietary supplements	65	3.7
4	Physical therapy	55	3.1
5	Massage	53	3.0
6	Acupuncture/moxibustion	36	2.0
7	Dietary therapy	32	1.8
8	Yoga	20	1.1
9	Chiropractic	16	0.9
10	Heat therapy	15	0.9
11	Bone setting (seitai)	13	0.7
12	Hot spring therapy	13	0.7
13	Magnetic therapy	12	0.7
14	Music therapy	9	0.5
15	Qigong	4	0.2
16	Forest therapy	2	0.1
17	Spiritual therapy	2	0.1
18	Ayurveda	1	0.1
19	Fasting therapy	1	0.1
20	Homeopathy	1	0.1
23	Others	20	1.1
24	No use of CAM	1,346	76.6

^a) ^multiple answers.

**Table 3-2.** CAM not Provided/Advised by Physicians (n=3,208) ^a)^.

	Product/Treatment method	n	%
1	Dietary supplements	378	11.8
2	Massage	126	3.9
3	Over-the-counter Kampo	112	3.5
4	Physical therapy	97	3.0
5	Acupuncture/moxibustion	75	2.3
6	Yoga	70	2.2
7	Hot spring therapy	65	2.0
8	Chiropractic	30	0.9
9	Bone setting	27	0.8
10	Aromatherapy	24	0.7
11	Dietary therapy	16	0.5
12	Magnetic therapy	16	0.5
13	Music therapy	16	0.5
14	Herbal therapy	10	0.3
15	Heat therapy	10	0.3
16	Qigong	10	0.3
17	Forest therapy	9	0.3
18	Spiritual therapy	9	0.3
19	Fasting therapy	8	0.2
20	Ayurveda	3	0.1
21	Homeopathy	2	0.1
22	Others	8	0.2
23	No use of CAM	2,482	77.4

^a)^ multiple answers.

### 4. The content of dietary supplements

Among vitamins, the most commonly used CAM was vitamin C (10.6%), followed by multivitamins (7.9%). Calcium (6.3%), iron (6.0%), and zinc (5.1%) were the most commonly used minerals. In addition, among “plant-type” supplements, blueberries (8.9%) and green juice (7.5%) were the most commonly used.

### 5. Use of Kampo medicines

Upon inquiring about the use of Kampo medicines, respondents had used either prescribed Kampo medicines (15.4%) or OTC Kampo medicines (15.7%) during the last 12 months (Table 4-1). We inquired about the name of the Kampo formula used (including both prescribed Kampo medicines and OTC Kampo medicines) from the 857 respondents who used Kampo medicines during the last 12 months (Table 4-2), and the formula kakkonto was the most commonly used (40.4%).

**Table 4-1.** Use of Kampo Medicines (n = 3,208).

		Used during the last 12 months		Currently using	
	Treatment Name	n	%	n	%
1	Over-the-counter Kampo medicines^ a)^	503	15.7	298	9.3
2	Prescribed Kampo medicines	494	15.4	275	8.6
3	No use of Kampo medicines (neither prescribed nor over-the-counter)	2,206	68.9	2,675	82.1

^a)^ Over-the-counter Kampo medicines refer to those self-purchased at a drugstore or pharmacy without a doctor’s prescription.

**Table 4-2.** Name of Kampo Formula Used during the last 12 months (n = 857) ^a)^.

	Name of Kampo formula	n	% in 857	% in 3,208
1	kakkonto	346	40.4	10.8
2	shoseiryuto	68	7.9	2.1
3	bakumondoto	50	5.8	1.6
4	shakuyakukanzoto	40	4.7	1.2
5	tokishakuyakusan	39	4.6	1.2
6	bofutsushosan	32	3.7	1.0
7	kamishoyosan	27	3.2	0.8
8	goreisan	22	2.6	0.7
9	keishibukuryogan	19	2.2	0.6
10	hochuekkito	17	2.0	0.5
11	rikkunshito	12	1.4	0.4
12	others	84	9.8	2.6
13	do not know/do not remember	299	34.9	9.3

^a)^ multiple answers.

### 6. Use of self-care methods

Self-care CAMs were used by 47.9% of respondents (non-users were 52% as in Table 5-1). The breakdown is as follows; “bath salts” (25.8%) and “walking” (25.3%) were the most commonly used self-care CAMs. The average 3-month frequency of use was high for “praying for health” (36.2 times), “bath salts” (28.2 times), and “music therapy” (26.7 times).

Regarding the reasons for the use of self-care, 17 of the 18 self-care items were utilized for health improvement, accounting for 65% or more of the self-care users (Table 5-2). For the degree of help for self-care, walking was considered helpful by 89.4% respondents, very helpful by 23.6% and somewhat helpful 65.8% respondents (Table 5-3).

**Table 5-1.** Use State and Use Frequency of CAM for Self-care ^a)^.

		n	%	Average 3-month use frequency (times)
1	Bath salt	828	25.8	28.0
2	Walking	812	25.3	22.4
3	Electric massage	195	6.1	12.3
4	Hot spring therapy	193	6.0	5.2
5	Yoga	137	4.3	10.7
6	Relaxation	95	3.0	4.1
7	Other health devices	78	2.4	17.8
8	Aromatherapy	61	1.9	11.6
9	Music therapy	50	1.6	26.7
10	Meditation	31	1.0	9.0
11	Forest therapy	28	0.9	3.3
12	Magnetic therapy	27	0.8	18.1
13	Praying for health	22	0.7	36.2
14	Qigong	17	0.5	6.6
15	Taijiquan	12	0.4	16.5
16	Art therapy	9	0.3	10.3
17	Heat therapy	9	0.3	8.0
18	Participation in healing associations	3	0.1	4.0
19	Others	30	0.9	
20	None	1,672	52.1	

^a)^multiple answers.

**Table 5-2.** Participant’s Reasons for Using CAM.

		No. of Use	Acute illness (less than 1 month)		Long-term illness (1 month or more)		For health improvement		Others	
		n	N	%	n	%	N	%	n	%
1	Bath salt	828	8	1.0	9	1.1	684	82.0	127	15.3
2	Walking	812	2	0.2	23	2.8	741	91.3	46	5.7
3	Electrical massage equipment	195	13	6.7	11	5.6	161	82.6	10	5.1
4	Hot spring therapy	193	1	0.5	10	5.2	164	85.0	18	9.3
5	Yoga	137	1	0.7	6	4.4	122	89.1	8	5.8
6	Relaxation	95	3	3.2	6	6.3	83	87.4	3	3.2
7	Other health devices	78	2	2.6	6	7.7	66	84.6	4	5.1
8	Aromatherapy	61	2	3.3	3	4.9	49	80.3	7	11.5
9	Music therapy	50	2	4.0	4	8.0	39	78.0	5	10.0
10	Meditation equipment	31	2	6.5	2	6.5	24	77.4	3	9.7
11	Forest therapy	28	1	3.6	2	7.1	22	78.6	3	10.7
12	Magnetic therapy	27	0	0.0	6	22.2	20	74.1	1	3.7
13	Praying for health	22	0	0.0	4	18.2	16	72.7	2	9.1
14	Qigong	17	0	0.0	2	11.8	15	88.2	0	0.0
15	Taijiquan	12	0	0.0	1	8.3	9	75.0	2	16.7
16	Art therapy	9	0	0.0	2	22.2	6	66.7	1	11.1
17	Heat therapy	9	1	11.1	5	55.6	3	33.3	0	0.0
18	Participation in healing associations	3	0	0.0	1	33.3	2	66.7	0	0.0

**Table 5-3.** Degree of Perceived Helpfulness of CAM Use.

			Very helpful		Somewhat helpful		Not helpful		Do not Know	
		n	n	%	n	%	n	%	n	%
1	Bath salt	828	104	12.6	533	64.4	81	9.8	110	13.3
2	Walking	812	192	23.6	534	65.8	33	4.1	53	6.5
3	Electrical massage equipment	195	32	16.4	145	74.4	12	6.2	6	3.1
4	Hot spring therapy	193	42	21.8	123	63.7	3	1.6	25	13.0
5	Yoga	137	29	21.2	95	69.3	10	7.3	3	2.2
6	Relaxation	95	21	22.1	63	66.3	9	9.5	2	2.1
7	Other health devices	78	14	17.9	51	65.4	4	5.1	9	11.5
8	Aromatherapy	61	17	27.9	40	65.6	2	3.3	2	3.3
9	Music therapy	50	18	36.0	31	62.0	0	0.0	1	2.0
10	Meditation	31	7	22.6	21	67.7	0	0.0	3	9.7
11	Forest therapy	28	8	28.6	15	53.6	3	10.7	2	7.1
12	Magnetic therapy	27	1	3.7	20	74.1	3	11.1	3	11.1
13	Praying for health	22	10	45.5	6	27.3	2	9.1	4	18.2
14	Qigong	17	5	29.4	9	52.9	3	17.6	0	0.0
15	Taijiquan	12	4	33.3	8	66.7	0	0.0	0	0.0
16	Art therapy	9	3	33.3	4	44.4	2	22.2	0	0.0
17	Heat therapy	9	2	22.2	6	66.7	1	11.1	0	0.0
18	Participation in healing associations	3	0	0.0	2	66.7	1	33.3	0	0.0

## Discussion

To our knowledge, this is the first study investigating the use of CAM applying the I-CAM-Q and using an internet survey in Japan. For CAM provided by healthcare providers during the last 12 months, the following therapies and products were used: Kampo medicines (OTC Kampo medicines: 15.7%; prescribed Kampo medicines: 15.4%), dietary supplements 11.8%, massage services 3.9%, and physical therapy 3.5%. Regarding the use of self-care methods during the last 12 months, the following methods and products were used: bath salts 25.8% and walking 25.3%.

Marui et al. conducted an internet survey on the CAM use of 3,227 members of the general population in Japan in February 2011 and found that dietary supplements were the most commonly used CAM (30.1%), followed by massage (7.9%), in the past one month ^(15)^. Although their study also used an internet survey, their questions covered data from the previous one month, whereas our questions covered data during the last 12 months. Moreover, our study can be used for future international comparisons using the I-CAM-QAs for the differences between I-CAM-Q and I-CAM-QJ, we did not change the basic structure of four parts and the survey on the past 12 months, added CAMs used in Japan such as Kampo medicines as herbal medicine, and deleted items not realistic in Japan such as herbalist as healthcare provider. Questionnaires were validated with pilot interview surveys and conducted an internet survey using them.

An internet survey on CAM use by Wemrell et al. from Sweden ^(18)^, reported71% of the respondents had used some form of CAM during the last 12 months. The most common types of CAM (53%) were natural remedies, such as herbal medicines and dietary supplements. Visits to CAM providers were most commonly rated as very helpful (70%). Therefore, the prevalence of CAM use is much higher in southern Sweden than in Japan, but the types of commonly used CAMs are similar. Although there was a survey on CAM use with the I-CAM-Q, various problems such as layout, difference in the definition and kinds of common CAMs among countries, were identified ^(19)^. Our study not only deepened our understanding of CAMs provided/advised by physicians, but also it surveyed CAM product types and self-care types used in Japan. Although the present study did not perform an international comparison, our results can be a basis for future international comparisons using the common tool (I-CAM-Q).

Dietary supplements are not covered by the national health insurance in Japan, and those CAMs are provided by physicians in private clinics at separate facilities. In Japan, such clinics are very rare. In contrast, approximately one-third of those surveyed had not received any health services during the last 12 months. However, cases in which CAM was purchased on the internet were also included in this group. There were similar cases in which CAM was purchased exclusively based on internet information and was used without professional advice. To take safety into consideration, warnings and countermeasures for such cases are important.

Acupuncture, moxibustion, and massage treatments are covered by the national health insurance on the condition that a physician issues a diagnosis document. This background is likely a common reason for the frequent use of physical therapy, massage, acupuncture, and moxibustion. In Japan, physicians rarely perform acupuncture, although they are legally allowed to do so. Instead, there are acupuncturists, who can run private clinics without physicians. Massage and related therapies are more commonly used than acupuncture.

In Japan, kakkonto is estimated to represent the most frequently used Kampo formula ^(20)^. Therefore, kakkonto is synonymous with Kampo medicine. When kakkonto is administered, patients typically remember the prescription name. However, when other Kampo formulas are administered, there are many cases in which the prescription name is not remembered. The section denoted by “do not know/do not remember” may include Kampo users, and one limitation of surveys using these types of questions involves the ability to recall the exact name of the Kampo formula.

Regarding orally administered treatments, such as herbs, vitamins, homeopathy, and dietary supplements, direct international comparisons are difficult due to differences in the survey/interviewing methods. Homeopathy is rarely used in Japan compared to Europe. It is reported that the rates of homeopathy use in European countries such as Denmark, Finland, Germany, Italy, Norway, Spain, Sweden, and the UK were 2-27% ^(21)^.

Interestingly, patients prefer CAM by various therapists/practitioners due to human relationships. As we previously reported ^(6)^, these CAM treatments are often prescribed for patients suffering from bone, joint, or muscular diseases who are dissatisfied with treatments by ordinary orthopedic surgeons or for patients whose symptoms are not severe enough to go to hospital, yet persist. Close listening and mindful conversation during treatment session would increase the patient’s satisfaction level.

Regarding self-care, the overall rates of self-care use in Japan were lower than those in the EU. For the new items added to the Japanese version of the survey (I-CAM-QJ), high use rates were noted for bath salts and walking. A health culture using baths (bath salts and hot spring therapy) is deeply rooted in Japan. In a survey by Fukuda et al., the rate of individuals with experience using hot springs was high ^(5)^.

Lee JA, et al. ^(16)^ published an internet survey using a Korean version of I-CAM-Q (I-CAM-QK) in 2018. The model of I-CAM-QK is a Japanese version of I-CAM-Q (I-CAM-QJ), which is potentially applied to non-Western region such as East Asia. On the other hand, there was no need to increase healthcare providers in Japan where ordinary physicians prescribe Kampo medicines under the unified medical licensing system, whereas Korean medical doctor was added as a healthcare provider in Korea. Furthermore, a survey is being conducted in Taiwan using a Taiwanese version of I-CAM-Q (I-CAM-QT). One of major CAM in these surveys would show to be of Chinese medicine origin. Current disease structures are not so different among Japan, Korea, and Taiwan. Further studies are warranted to elucidate the differences in the name and usage of Kampo formulas or Chinese patent drugs/ Chinese formula in decoction form in certain diseases and semi-healthy conditions among these countries/areas. Moreover, how cultural and social determinants of health are involved in the above differences would be discussed including the possible use of health insurance reimbursement data after the Taiwanese survey results are published.

There are two limitations of our study. First, we could not identify who was using any particular type of CAM or whether some people used numerous types of CAMs while others used little or no CAMs. However, the higher than reported rate of CAM users may be present because people who were interested in the topic of CAM were more likely to answer a questionnaire than those who were not interested, in internet survey, in particular. Therefore, the internet survey has a high risk of selection bias, and the generalizability of this survey may be questionable. Second, we did not directly compare the two questionnaires (I-CAM-QJ and direct translation version of I-CAM-Q) within the same person or within the same population. Since we have not yet verified criterion-related validity, modified I-CAM-Q should be used with caution, and additional studies should be conducted to show the validity.

### Conclusions

An internet survey on CAM use by the general Japanese population with a modified I-CAM-Q (I-CAM-QJ) revealed that Kampo medicines and dietary supplements were the most commonly used CAMs in Japan. Because some CAMs were purchased based on internet information and used without professional advice, warnings, and countermeasures for such cases are desirable for the safe use of CAM.

## Article Information

### Conflicts of Interest

None

### Sources of Funding

This work was supported by the AMED (Japan Agency for Medical Research and Development) grant number JP15lk0310008h0001.

### Acknowledgement

We would like to thank Dr. Naoki Nago (Tokyo), Dr. Jiro Imanishi (Kyoto), and Dr. Satoko Kishida (Kyoto) for their cooperation during the pilot interview survey prior to the internet survey. The authors would also like to acknowledge the editors of the manuscript from the Springer Nature Author Services, authorservices.springernature.com.

### Author Contributions

All authors constructed and revised the questionnaire. YM and KT developed the study concept. YM and KY analyzed the data and wrote the manuscript. IA and KH acquired and analyzed the data and reviewed the manuscript. KY contributed to the work-related statistics and reviewed and revised the manuscript. All authors read and approved the final manuscript.

### Approval by Institutional Review Board (IRB)

Approval code: no. 168 (21 July 2015) from Tokyo Ariake University of Medical and Health Sciences.

## Supplement

Supplementary Material 1
