# Supplementary material for: Use of Complementary and Alternative Medicine in Japan: A Cross-sectional Internet Survey Using the Japanese Version of the International Complementary and Alternative Medicine Questionnaire
Source: JMA J. 2019 Mar 4;2(1):35–46. doi: 10.31662/jmaj.2018-0044 (PMC7930716; doi:10.31662/jmaj.2018-0044)
Supplement: Supplementary file 1 — Supplementary Material 1 [file 2433-3298-2-1-0035-s001.pdf]

A questionnaire in relation to medical care / health services. (I-CAM-QJ)

The purpose of this survey is to gather more detail around how people are using our medical care and health services.
We ask that those people who are not medical professionals (hospital and clinic employees, pharmacists and so on), involved in advertising or the media, or the market research industry please participate in this survey.

This survey involves questions about your own personal health, and also the hospital itself.
This questionnaire is composed from four questions:
1) Health care providers
2) Medical care / health services received from physicians
3) Dietary Supplements
4) Self-help practices

English translation from the final version of the Japanese questionnaire
March 2016

※If you have used a particular service, please tick the box entitled 'Yes', If you have not used a particular service, or you don't know, please tick 'No' and go to the next question

Question 0. Questions about your own

Date（MM/DD/YYYY）

Please check / fill out this questionnaire.

Q1. What is your date of birth? **（MM/DD/YYYY）**

Q2. Are you male or female? **(1. Male　２. Female)**

Q3. What is your final academic background?（Check only one）

（1. Middle school　2. High school　3. Special college　4. Upper Secondary Specialized Training School
5. Junior College　6. University　7. Graduate school　8. Others (other specify : ________________)　）

Q4. How is your general health condition?（Check only one）

（　1. Very good　2. Good　3. Acceptable　4. Bad　5. Very Bad　）

Q5. Do you have a longtime disease or disorder? (‘a longtime’ means that you have the symptom more than one month)
（　１. Yes　２. No　）

↓

Q6. If you YES, which diseases ?（Check all that apply）

1. Hypertension　2. Stroke (cerebral hemorrhage, cerebral infarction, etc.)　3. Heart disease　4. Diabetes　5. Dyslipidemia (hyperlipidemia)
6. Respiratory illness　7. Diseases of the gastrointestinal tract (gastrointestinal, liver, gall bladder, pancreas, etc.)
8. Kidney and urological diseases　9. Musculoskeletal diseases (osteoporosis, arthropathy, back pain, etc.)　10. Trauma (falls, fractures, etc.)
11. Cancer (including blood cancer and sarcoma)　12. Blood disease (other than tumor)　13. Immune disease (such as collagen disease)
14. Mental disorders such as depression / dementia　15. Nose disease　16. Eye disease　17. Ear disease　18. Skin disease
19. Tooth disease　20. Others（other specify :　　　　　　　　　　　　）

Q7.　Do you have a private medical insurance ? **（　１. Yes　２. No　）**

※If you have used a particular service, please tick the box entitled 'Yes', If you have not used a particular service, or you don't know, please tick 'No' and go to the next question

Question １. Health care providers

Have you seen any of the following providers **in the last 12 months**?		Number of times you saw this provider **in the last 3 months**?	Please indicate the **main** reason you **last** saw the provider. (Check only one)					How helpful was it for you to see this provider? (Check only one)			
			1 For an acute illness / condition, one that lasted less than one month	2 To treat a long-term health condition(one that lasted more than one month) or its symptoms	3 To improve well-being	4 Other	(Please specify the other reason)	1 Very	2 Somewhat	3 Not at all	4 Don't know
**Physician**	Yes・No	times	1	2	3	4		1	2	3	4
**Dentist**	Yes・No	times	1	2	3	4		1	2	3	4
**Pharmacist**	Yes・No	times	1	2	3	4		1	2	3	4
**Nurse / Public Health Nurse**	Yes・No	times	1	2	3	4		1	2	3	4
**Maternity nurse**	Yes・No	times	1	2	3	4		1	2	3	4
**Massage practitioner / Acupressure therapist**	Yes・No	times	1	2	3	4		1	2	3	4
**Acupuncturist/ Moxibustionist**	Yes・No	times	1	2	3	4		1	2	3	4
**Judo therapist (Bonesetter)**	Yes・No	times	1	2	3	4		1	2	3	4
**Nutritionist**	Yes・No	times	1	2	3	4		1	2	3	4
**Yoga instructor**	Yes・No	times	1	2	3	4		1	2	3	4
**Chiropractor**	Yes・No	times	1	2	3	4		1	2	3	4
**Manual therapist**	Yes・No	times	1	2	3	4		1	2	3	4
**Aromatherapist / Herb therapist**	Yes・No	times	1	2	3	4		1	2	3	4
**Spiritual therapist**	Yes・No	times	1	2	3	4		1	2	3	4
**Homeopathy therapist**	Yes・No	times	1	2	3	4		1	2	3	4
**Other (please specify):**	Yes・No	times	1	2	3	4		1	2	3	4
**Other (please specify):**	Yes・No	times	1	2	3	4		1	2	3	4

※If you have used a particular service, please tick the box entitled 'Yes', If you have not used a particular service, or you don't know, please tick 'No' and go to the next question

Question 2. Medical care / health services received from physicians

If you not seen a physician in the past 12 months, please go to question 3.

Have you received any of the following medical care or health services from physicians **in the last 12 months**?		Number of times you saw this provider **in the last 3 months**?	Please indicate the **main** reason you **last** received the care / service. (Check only one)					How helpful was it to receive you care / service from the physician? (Check only one)			
			1 For an acute illness / condition, one that lasted less than one month	2 To treat a long-term health condition(one that lasted more than one month) or its symptoms	3 To improve well-being	4 Other	(Please specify the other reason)	1 Very	2 Somewhat	3 Not at all	4 Don't know
**Acupuncture and moxibustion**	Yes・No	times	1	2	3	4		1	2	3	4
**Massage**	Yes・No	times	1	2	3	4		1	2	3	4
**Dietary supplement**	Yes・No	times	1	2	3	4		1	2	3	4
**Aromatherapy**	Yes・No	times	1	2	3	4		1	2	3	4
**Herb therapy**	Yes・No	times	1	2	3	4		1	2	3	4
**Homeopathy**	Yes・No	times	1	2	3	4		1	2	3	4
**Spiritual therapy**	Yes・No	times	1	2	3	4		1	2	3	4
**Music therapy**	Yes・No	times	1	2	3	4		1	2	3	4
**Spa therapy**	Yes・No	times	1	2	3	4		1	2	3	4
**Ayurveda**	Yes・No	times	1	2	3	4		1	2	3	4
**( )**	Yes・No	times	1	2	3	4		1	2	3	4
**( )**	Yes・No	times	1	2	3	4		1	2	3	4
**( )**	Yes・No	times	1	2	3	4		1	2	3	4

※If you have used a particular service, please tick the box entitled 'Yes', If you have not used a particular service, or you don't know, please tick 'No' and go to the next question

Question 3. Use of Dietary Supplements
(Dietary Supplements, Health Foods, Kampo Medicine, etc.)

《 Vitamins / Minerals 》 Have you used vitamins / minerals **in the last 12 months**?							(Yes・No)			
e.g.）Vitamin A, Vitamin B1, Vitamin B2, Vitamin B6, Vitamin B12, Vitamin C, Vitamin D, Vitamin E, Vitamin K, Multiple vitamin, Pantothenic acid, Biotin, Niacin, Folic acid, Iron, Calcium, Copper, Zinc, Magnesium, Potassium, Multi-mineral etc.										
Please add the product name used each in the last 12 months.<br>（Regardless of the physician's prescription or its advise）	Do you **currently** use the product?	Please indicate the **main** reason that applies to your **last** use. (Check only one)					How helpful did you find this product? (Check only one)			
		1<br>For an acute illness / condition, one that lasted less than one month	2<br>To treat a long-term health condition(one that lasted more than one month) or its symptoms	3<br>To improve well-being	4<br>Other	(Please specify the other reason)	1<br>Very	2<br>Somewhat	3<br>Not at all	4<br>Don't know
( )	Yes・No	1	2	3	4		1	2	3	4
( )	Yes・No	1	2	3	4		1	2	3	4
**《Diet treatment》 《Medicinal liquor》**		**(Yes・No)**								
**Macrobiotic**	Yes・No	1	2	3	4		1	2	3	4
**Fasting therapy**	Yes・No	1	2	3	4		1	2	3	4
**Low carbohydrate diet**	Yes・No	1	2	3	4		1	2	3	4
( )	Yes・No	1	2	3	4		1	2	3	4
( )	Yes・No	1	2	3	4		1	2	3	4
( )	Yes・No	1	2	3	4		1	2	3	4
**Alcohol containing natural ingredients**	Yes・No	1	2	3	4		1	2	3	4
( )	Yes・No	1	2	3	4		1	2	3	4
( )	Yes・No	1	2	3	4		1	2	3	4
**《Homeopathy》** Have you used the homeopathy product **in the last 12 months**?<br>If you YES, please add the product name and answer questions in below.							**(Yes・No)**			
( )	Yes・No	1	2	3	4		1	2	3	4
( )	Yes・No	1	2	3	4		1	2	3	4
**《Kampo Medicine》**<br>Kampo Medicine is classified as follows:										

※If you have used a particular service, please tick the box entitled 'Yes', If you have not used a particular service, or you don't know, please tick 'No' and go to the next question

(1) ETC: Manufactured traditional Kampo medicines for prescription
(2) OTC: OTC Kampo medicines
1. Have you used these Kampo Medicines **in the last 12 months**? 2. Do you **currently** use these Kampo Medicines?
* If you YES, please check in below questions. If you NO, please go to 《 **Dietary supplements, Health foods** 》 questions.

(1) Manufactured Kampo medicines for prescription	1. I used it in the last 12 months（**Yes・No**） → 2. Using currently（**Yes・No**）
(2) OTC Kampo medicines (which are able to purchase own at drugstore without prescription)	1. I used it in the last 12 months（**Yes・No**） → 2. Using currently（**Yes・No**）

Please indicate the Kampo Medicine Product you used.
e.g.） kakkonto, kamishoyosan, keishibukuryogan、goreisan, shakuyakukanzoto, shoseiryuto, tokishakuyakusan, bakumondoto, hochuekkito, bofutsushosan, rikkunshito, etc.

Please add the name of used Kampo medicine.	Do you **currently** use the product?	Please indicate the **main** reason you **last** used this product. (Check only one)					How helpful did you find the product? (Check only one)			
		1 For an acute illness / condition, one that lasted less than one month	2 To treat a long-term health condition(one that lasted more than one month) or its symptoms	3 To improve well-being	4 Other	(Please specify the other reason)	1 Very	2 Somewhat	3 Not at all	4 Don't know
( )	Yes・No	1	2	3	4		1	2	3	4
( )	Yes・No	1	2	3	4		1	2	3	4
《**Dietary supplements, Health foods**》 e.g.） Glucosamine, Chondroitin, Saw palmetto, Green juice, Collagen, Rooibos tea, Placenta, Blueberry etc.										
**Glucosamine**	Yes・No	1	2	3	4		1	2	3	4
**Chondroitin**	Yes・No	1	2	3	4		1	2	3	4
**Saw palmetto**	Yes・No	1	2	3	4		1	2	3	4
**Green juice**	Yes・No	1	2	3	4		1	2	3	4
( )	Yes・No	1	2	3	4		1	2	3	4
( )	Yes・No	1	2	3	4		1	2	3	4

《**Purchase place**》 Where did you buy the product? （Check all that apply）	1. Pharmacy(Drug store) 2. Supermarket 3. Internet shopping 4. Mail order 5. Others（ ）

※If you have used a particular service, please tick the box entitled 'Yes', If you have not used a particular service, or you don't know, please tick 'No' and go to the next question

Question 4. Self-help practices

This is a last Question. Thank you for your cooperation.

Have you used any of the following self-help practices **in the last 12 months**? （Regardless of the physician's prescription or its advise.）		Number of times you used practice **in the last 3 months**?	Please indicate the **main** reason that applies to your **last** use of the self-help practice. (Check only one)					How helpful did you find the self-help practice? (Check only one)			
			1 For an acute illness / condition, one that lasted less than one month	2 To treat a long-term health condition(one that lasted more than one month) or its symptoms	3 To improve well-being	4 Other	(Please specify the other reason)	1 Very	2 Somewhat	3 Not at all	4 Don't know
**Meditation**	Yes・No	times	1	2	3	4		1	2	3	4
**Yoga**	Yes・No	times	1	2	3	4		1	2	3	4
**Qigong**	Yes・No	times	1	2	3	4		1	2	3	4
**Tai Chi**	Yes・No	times	1	2	3	4		1	2	3	4
**Relaxation techniques**	Yes・No	times	1	2	3	4		1	2	3	4
**Music therapy**	Yes・No	times	1	2	3	4		1	2	3	4
**Picture therapy**	Yes・No	times	1	2	3	4		1	2	3	4
**Attend traditional healing ceremony**	Yes・No	times	1	2	3	4		1	2	3	4
**Praying for own health**	Yes・No	times	1	2	3	4		1	2	3	4
**Electric massage machine**	Yes・No	times	1	2	3	4		1	2	3	4
**Other health appliances**	Yes・No	times	1	2	3	4		1	2	3	4
**Walking**	Yes・No	times	1	2	3	4		1	2	3	4
**Forest therapy**	Yes・No	times	1	2	3	4		1	2	3	4
**Aromatherapy**	Yes・No	times	1	2	3	4		1	2	3	4
**Hyperthermia**	Yes・No	times	1	2	3	4		1	2	3	4
**Magnet therapy**	Yes・No	times	1	2	3	4		1	2	3	4
**Spa therapy**	Yes・No	times	1	2	3	4		1	2	3	4
**Bath additive**	Yes・No	times	1	2	3	4		1	2	3	4
**( )**	Yes・No	times	1	2	3	4		1	2	3	4
**( )**	Yes・No	times	1	2	3	4		1	2	3	4

※If you have used a particular service, please tick the box entitled 'Yes', If you have not used a particular service, or you don't know, please tick 'No' and go to the next question
